
JOURNAL INFORMATION
==============================
NLM Title Abbreviation: Radiol Adv
Journal ID: Radiol Adv
NLM Journal ID: 9918840888906676
Journal ID: radadv

Radiology Advances

EISSN: 2976-9337
Publisher: Oxford University Press

ARTICLE INFORMATION
==============================
PMCID: PMC13537815
PMID: 42688962
DOI: 10.1093/radadv/umag016
Article ID: umag016
Article version: 1
Subjects: Art of Imaging, AcademicSubjects/MED00870

The diagnostic symphony

https://orcid.org/0000-0002-5848-7072 Singh Yashbir ME, PhD Department of Radiology, Mayo Clinic, Rochester, MN 55905, United States

https://orcid.org/0000-0003-3151-009X Gores Gregory J MD Division of Gastroenterology and Hepatology, Mayo Clinic, Rochester, MN 55905, United States

Corresponding author: Yashbir Singh, ME, PhD, Department of Radiology, Mayo Clinic, 200 1st St SW, Rochester, MN 55905, United States (Singh.yashbir@mayo.edu)
Publication date: 2026 Sep
Electronic publication date: 2026 Sep 2
Volume: 3
Issue: 5
Electronic Location ID: umag016
Received 2026 Mar 6; Revised 2026 Mar 13; Accepted 2026 Mar 18
Copyright: © The Author(s) 2026. Published by Oxford University Press on behalf of the Radiological Society of North America.
Copyright year: 2026
License: This is an Open Access article distributed under the terms of the Creative Commons Attribution License (https://creativecommons.org/licenses/by/4.0/), which permits unrestricted reuse, distribution, and reproduction in any medium, provided the original work is properly cited.
License URL: https://creativecommons.org/licenses/by/4.0/

Page count: 2

==============================
Supplementary Material

umag016_Supplementary_Data

Supplementary material

Supplementary material is available at Radiology Advances online.

Conflicts of interest

Please see ICMJE form(s) for author conflicts of interest. These have been provided as supplementary materials.

Caption: Where science meets soul—the human heart, painted in the language of art and anatomy.

For image description, please refer to the figure legend and surrounding text

Yashbir Singh, ME, PhD, Assistant Professor, Radiology, Mayo Clinic, Rochester, MN, USA

For image description, please refer to the figure legend and surrounding text

Gregory J. Gores, MD, Professor, Division of Gastroenterology and Hepatology, Mayo Clinic, Rochester, MN, USA

For image description, please refer to the figure legend and surrounding text

